# Comparative transcriptomic analyses revealed divergences of two agriculturally important aphid species

**DOI:** 10.1186/1471-2164-15-1023

**Published:** 2014-11-25

**Authors:** Dahai Wang, Qi Liu, Huw D Jones, Toby Bruce, Lanqin Xia

**Affiliations:** Institute of Crop Sciences /The National Key Facility for Crop Gene Resource and Genetic Improvement, Chinese Academy of Agricultural Sciences (CAAS), 12 Zhongguanchun South Street, Beijing, 10081 China; Beijing Autolab Biotechnology Co. Ltd, Beijing Jiaotong University, Eastern Campus, Beijing, 100081 China; The Medical College of Wenzhou, Wenzhou, 325032 China; Rothamsted Research, Harpenden, Hertfordshire, AL5 2JQ UK

**Keywords:** Grain aphid, Pea aphid, Transcriptome, Comparative transcriptomic analysis, Divergence

## Abstract

**Background:**

Grain aphid (*Sitobion avenae* F) and pea aphid (*Acyrthosiphon pisum*) are two agriculturally important pest species, which cause significant yield losses to crop plants each year by inflicting damage both through the direct effects of feeding and by vectoring debilitating plant viruses. Although a close phylogenetic relationship between grain aphid and pea aphid was proposed, the biological variations between these two aphid species are obvious. While the host ranges of grain aphid is restricted to cereal crops and in particular wheat, that of pea aphid is wider, mainly colonizing leguminous plant species. Until now, the genetic factors underlying the divergence between grain aphid and pea aphid still remain unclear due to the limited genomic data of grain aphid available in public databases.

**Results:**

Based on a set of transcriptome data of grain aphid generated by using Roche 454 GS-FLX pyrosequencing, comparative analysis between this set of transcriptome data of grain aphid and mRNA sequences of pea aphid available in the public databases was performed. Compared with mRNA sequences of pea aphid, 4,857 unigenes were found to be specifically presented in the transcriptome of grain aphid under the rearing conditions described in this study. Furthermore, 3,368 orthologous pairs which could be calculated with both nonsynonymous (Ka) and synonymous (Ks) substitutions were used to infer their sequence divergences. The average differences in the coding, 5′ and 3′ untranslated regions of these orthologs were 10.53%, 21.29% and 18.96%, respectively. Moreover, of 340 orthologs which were identified to have evolved in response to positive selection based on the rates of Ka and Ks substitutions, 186 were predicted to be involved in secondary metabolism and xenobiotic metabolisms which might contribute to the divergence of these two aphid species.

**Conclusions:**

The comprehensive transcriptome divergent sequence analysis between grain aphid and pea aphid provides an invaluable resource for the investigation of genes involved in host plant adaptation and evolution. Moreover, the demonstration of divergent transcriptome sequences between grain aphid and pea aphid pave the way for the investigation of the molecular mechanisms underpinning the biological variations of these two agriculturally important aphid species.

**Electronic supplementary material:**

The online version of this article (doi:10.1186/1471-2164-15-1023) contains supplementary material, which is available to authorized users.

## Background

Aphids are major agricultural pests which cause significant yield losses to crop plants each year by inflicting damage both through the direct effects of feeding and by vectoring debilitating plant viruses [1]. Annual worldwide crop losses due to aphids are estimated at hundreds of millions of dollars [2]. The application of nitrogen fertilizer and elevation of atmospheric CO_2_ concentration exacerbate aphid infestation [3]. The major aphid species infesting wheat in China are the grain aphid (*Sitobion avenae* F.), greenbug (*Schizaphis graminum* Rondani), bird-cherry oat aphid (*Rhopalosiphum padi* Linnaeus) and rose-grain aphid (*Metopolophium dirhodum* Walker). Of these, grain aphid is the most dominant and destructive one, affecting most of the wheat production areas [4]. The grain aphid is also a major pest of wheat in Europe and North America [5]. In some wheat production areas, it has damaging infestations every year, causing the wide-spread use of chemicals and as much as 15 to 60% of severe reduction in wheat yield [6]. Due to the complexity of plant-aphid interactions and the rapid development of resistant pest biotypes, outbreak of aphids causing substantial losses of wheat are reported regularly [5, 6].

Aphids are important agricultural pests and also biological models for study of insect-plant interactions, virus vectoring and host plant adaptation [1]. Recently, the release of genome sequence of the pea aphid provided a foundation for post-genomic studies of fundamental biological questions both in pea aphid and other aphid species. It revealed the presence of more coding genes than in previously sequenced insects and in particular, the presence of genes with no orthologs in other insects [1]. Now, the assembled genome sequence data, ESTs and full length cDNAs of the pea aphid are accessible at the AphidBase web portal (http://www.aphidbase.com) [7]. With the release of the first aphid genome and the general accessibility of next generation sequencing technologies, there is an expectation within the aphid research community that we have reached a tipping point for genome information from additional aphid species and high-resolution comparative genomic and evolutionary analyses [8]. Although a close phylogenetic relationship between pea aphid and grain aphid was illustrated based on analysis of partial sequence of EF1 alpha orthologs in different aphid species [8], the diversity between these two aphid species is obvious. For example, the host ranges of grain aphid are restricted to cereal crops and in particular wheat, that of pea aphid is wider, mainly colonizing many leguminous plant species. Until now, the molecular mechanisms underlying their divergence and host adaptation have not been documented due to the lack of enough genetic data of grain aphid available in the public databases. In a previous study, we performed *de novo* transcriptome assembly and gene expression analyses of the alimentary canals of grain aphids before and after feeding on wheat plants by using Illumina RNA sequencing [9]. However, a complete transcriptome and/or genomic data of the grain aphid have not been documented so far, in spite of the fact that grain aphid is most dominant and destructive aphid pest affecting wheat production in China, Europe and North America.

Next-generation sequencing (NGS) technologies allow direct sequencing of cDNA generated from messenger RNA (RNA-seq) [10]. These new technologies enable the de novo reconstruction of the transcriptome for a non-model organism [11], leading to novel opportunities for expression profiling of organisms lacking any genome or transcriptome sequence information [11, 12]. In this paper, in order to reveal the genetic factors underlying the divergence between the grain and pea aphids, comparative analysis between the transcriptome data of grain aphid generated in our lab by Roche 454 GS-FLX pyrosequencing and mRNA sequences of pea aphid available in the public databases was performed. A number of orthologous genes that showed signs of diversifying natural selection were identified. This comparative analysis provides an invaluable resource for the investigation of genes involved in plant infestation, host adaptation and insecticide resistance. Moreover, the demonstration of divergent transcriptome sequences between grain aphid and pea aphid pave the way for the investigation of the molecular mechanisms underpinning the biological variations of these two destructive aphid species.

## Results

### Roche 454 GS-FLX pyrosequencing and assembly of grain aphid transcriptome

**Figure 1:**
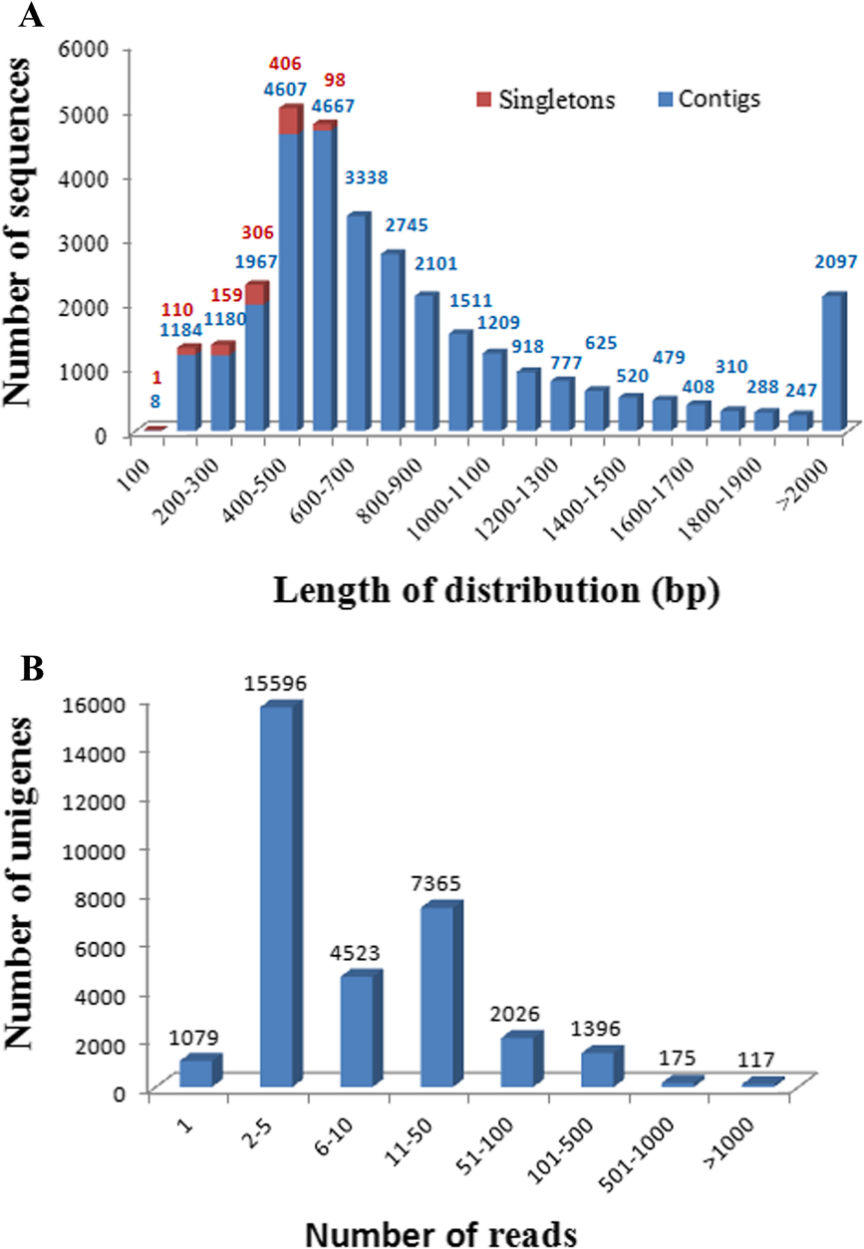
**Length distribution of contigs, singletons and unigenes and the distribution of reads number of the assembled unigenes. (A)** The length distributions of contigs, singletons and unigenes. **(B)** The reads distribution of unigenes.

To obtain a global view of the grain aphid transcriptome, 300 grain aphids at different developmental stages, which were derived from a single clonal lineage, were used for RNA isolation. High-throughput RNA sequencing (RNA-seq) was performed with Roche 454 GS-FLX pyrosequencing platform. A total of 1,106,696 reads, with an average length of 380 bp, amounting to a total 401.7 Mb, were obtained and deposited in the NCBI Short Read Archive (SRA) under the accession number: SRA065628. The raw reads were assembled using Mira 3 assembly software packages (http://www.chevreux.org/projects_mira.html), and resulted in 44,682 contigs with an average length of 812.5 bp (Table 1). The un-assembled high quality reads which occupied 1.79% (16,677) of the total cleaned reads were added to the contigs dataset and generated in total of 61,359 sequences. These sequences were further assembled with gsAssembler v 2.3 software. Among 61,359 sequences, 29,679 were identified as unique sequences, while the remaining 31,680 sequences were re-assembled into 3,513 contigs. After removing sequences below 100 bp and transcripts of *Buchnera aphidicola* (an endosymbiont of aphids), we obtained 32,277 distinct sequences as unigenes which included 31,199 contigs and 1078 singletons (Table 1). The flow chart of data assembly was provided in the Additional file 1. The assembled transcriptome data were submitted and have been deposited at DDBJ/EMBL/GenBank under the accession GAPL00000000. The average coverage of the contigs was 15.5 x (which was calculated by dividing total length of assembled reads by length of all contigs), and the longest length and the average length of the assembled contigs were 11,206 bp and 866 bp, respectively (Table 1). Next, we analyzed the length distribution of the contigs, singletons, unigenes and the number of reads per unigene. As shown in Figure 1, 7,878 contigs (25.3%) were longer than 1000 bp, 14,362 contigs (46.0%) ranged from 500 bp to 1000 bp, whereas 8946 contigs (28.7%) were below 500 bp (Figure 1). The N50 and N90 of the contigs, were 1,021 and 479 bp, respectively (Table 1). The un-assembled high quality reads were assigned as singletons, which occupied 0.12% (1,078) of the total cleaned reads. The length of singletons varied from 100 bp to 600 bp with an average of 369 bp. The majority of singletons were between 200 bp and 600 bp in length, and 10.3% of them were below 200 bp (Figure 1). The number of reads per unigene varied from 1 to 13,917 with an average of 32.5. The group that consisted of less than 5 reads contained 16,674 unigenes, representing 51.7% of the total assembled unigenes. Whereas 117 unigenes had more than 1000 reads, 175 unigenes had 501 to 1000 reads, and 1,396 unigenes had 100 to 500 reads. Unigenes with reads below 100 represented 94.7% (30,588) of the total number of unigenes (Figure 1).

**Table 1:** **Summary of the transcriptome of grain aphid**

Items	Values
Total number of reads (bp)	1,106,696
Total base pairs (Mb)	401.7
Average read length (bp)	380
Total number of contigs	31,199
Singletons	1,078
Total unigenes	32,277
N50 of contigs (bp)	1,021
N90 of contigs (bp)	479
Average length of singletons (bp)	369
The longest length of contigs (bp)	11206
Average length of contigs (bp)	866
Average length of unigenes (bp)	850

The A, T, C and G proportions of the assembled unigenes were 30.02%, 30.42%, 19.81% and 19.64%, respectively. The GC content of the transcriptome data of grain aphid was 39.45%, a value slightly higher than that of the pea aphid which was 38.8% [1]. Of these 32,277 unigenes assembled, the average length of coding regions (CDSs) was 556 bp and the GC content of the CDSs was 42.39%. Among which, 17,256 unigenes, of which GC content was 31.73%, could be predicted with 3′UTRs and the average length of the 3′UTRs was 282.6 bp. At the same time, 16,846 unigenes were predicted with 5′UTRs, of which GC content was 36.42% and the average length of 5′UTRs was 262.3 bp. It was interesting to note that the GC contents among CDSs, 5′UTRs and 3′UTRs were different and the CDSs had highest GC content among these three categories, whereas the GC content of 5′UTRs was 4.69% higher than that of 3′UTRs.

### The functional annotation and classification of the assembled unigenes

Functional annotations of the derived unigenes were performed using Basic Local Alignment Search Tool (BLAST) against the public available databases such as National Center for Biotechnology Information (NCBI) Nucleotide Acid Database (Nt) and Non-redundancy Protein Database (Nr), and BLASTx against the Swiss-Prot Database. A total of 25,389 unigenes (78.7%) matched the Nt database with an E value of 1E-10, and 21,635 unigenes (67.9%) matched genes in Nr database with E value of 1E-5. BLASTx against Swiss-Prot Database indicated that 16,211 unigenes (50.2%) were annotated with an E-value of 1E-5 (Table 2, See Additional file 2).

**Table 2:** **Numbers of annotated unigenes in the public databases**

Annotation databases	Annotated unigene number	Percentage (%)
Nt	25,389	78.7
Nr	21,635	67.0
Swiss-Prot	16,211	50.2
KEGG	13,876	43.0
GO	11,731	36.3
KOG	15,957	49.4
Total assembled unigenes	32,277	

All unigenes that matched Swiss-Prot database entries were further classified by Gene Ontology (GO) terms according to the number of the matched entries. In total, 11,731 unigenes (36.3%) had GO terms (Table 2, See Additional file 2). Furthermore, the unigenes were also classified according to Eukaryotic Orthologous Groups (KOG) terms. In total, 15,957 unigenes (49.4%) had KOG terms (Table 2, See Additional file 2). In addition, 13,876 unigenes (43%) were mapped to the Kyoto Encyclopedia of Genes and Genomes (KEGG) pathways (Table 2) and grouped into 193 categories. The majority of classifications were metabolic pathways and biosynthesis of secondary metablolites, which represented 19.15% and 6.05% of total annotated unigenes, respectively.

### The transcriptomic divergences between grain aphid and pea aphid

Comparisons of the transcriptome data of different aphid species would provide useful information in deciphering many of the specialized biological variations underlying the role of aphids as plant pests, and in understanding the transcriptome evolution and the genetic factors underlying the divergence of these species. To compare the sequence divergence of these two aphid species, we analyzed the possible orthologous genes between the transcriptome of grain aphid obtained in this study and the pea aphid mRNA sequence data which are available in website (https://www.aphidbase.com/aphidbase/content/download/3250/33670/file/aphidbase_2.1b_mRNA.fasta.bz2) using bidirectional best hit which has been widely used to identify orthologous genes [13, 14].

**Figure 2:**
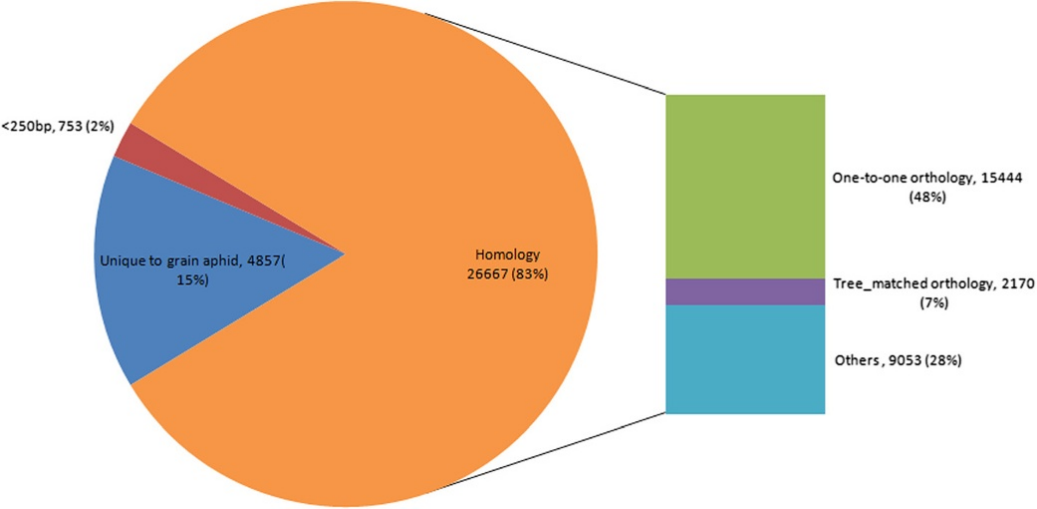
**Comparison of the assembled grain aphid unigenes with pea aphid mRNA sequences.** Among the 32,277 unigenes assembled, 26,667 grain aphid unigenes (yellow) were homologous to pea aphid mRNA sequences, of these, 15,444 were one-to-one orthologs (green), 2,170 were tree-matched orthologs (purple), and 9,053 were homologous to pea aphid mRNA sequences with the ratio of matched region below 50% and some paralogs (light blue). The number of unigenes which were unique to grain aphids compared with pea aphid mRNA was 4,857 (blue). And the remaining 753 sequences (red) which were shorter than 250 bp and had no homologous sequences with pea aphid mRNA identified by BLASTn tool were excluded because they were too short sequence for tBLASTx searching. All orthologs were identified using ETE pipeline with SOS of 0.0.

First, BLASTn and tBLASTx tools were employed to screen out unigenes homologous to pea aphid. Using BLASTn tool, 25,294 unignes homologous to pea aphid mRNA sequences were identified and 6,983 sequences un-matched were further subjected to comparison with pea aphid mRNA data at translation level. As sequences below 250 bp were too short to be translated or compared with the translated sequences from pea aphid mRNA, they were removed from the 6,983 sequences dataset, and finally 6,230 sequences remained. Furthermore, by using tBLASTx tool, we found that among 6,230 sequences, 1,114 sequences were orthologous to pea aphid mRNA sequences. For the remaining 5,116 sequences, 259 were annotated with predicted functions similar to these of sequences from pea aphid in Nt database. Thus, among the 32,277 unigenes assembled, 26,667 unigenes (25,294 + 1,114 + 259) which occupied 81.8% of total assembled unigenes showed homology to pea aphid mRNA sequences. Of these, 15,444 were one-to-one orthologs, 2,170 were tree-matched orthologs, and the remaining 9,053 were either homologous to pea aphid mRNA sequences with the ratio of matched region below 50% or some paralogs (Figure 2). During BLASTn analysis, 753 sequences which were shorter than 250 bp and had no homologous sequences with pea aphid mRNA identified by BLASTn tool were excluded because they were too short for tBLASTx searching. The remaining 4,857 unigenes (32,277-26,667-753) were identified to be grain aphid specific genes under the described rearing conditions in this study (Figure 2). The presence and expression of some of the grain aphid specific genes were confirmed by qRT-PCR with randomly selected 14 unigenes (Data not shown).

**Figure 3:**
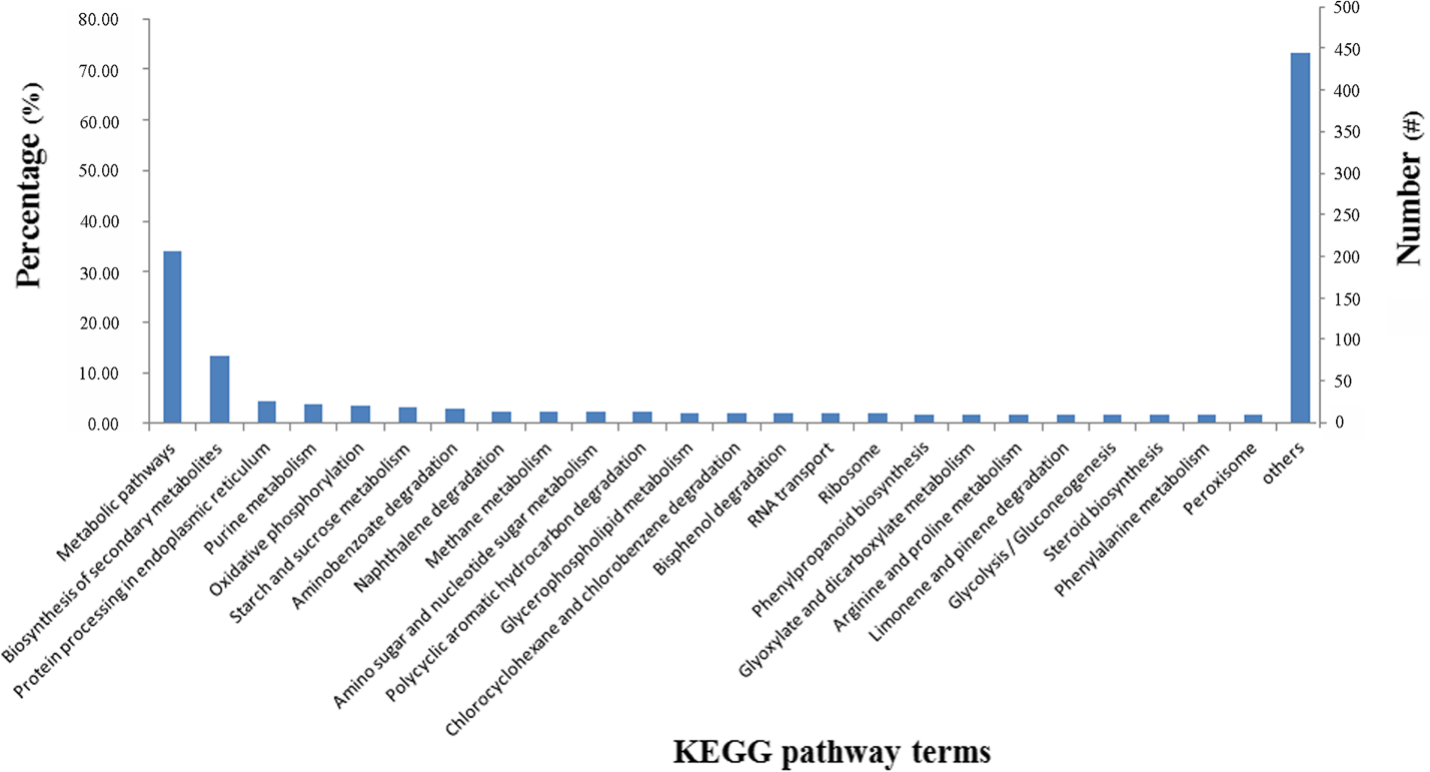
**The KEGG pathway classifications of the grain aphid specific unigenes.** Among 4,857 unigenes, 607 had been annotated with KEGG terms, and the classification of these unigenes showed that many of them were categorized into the groups of metabolic pathways (34.1%), biosynthesis of secondary metabolites (13.4%), protein processing in endoplasmic reticulum (4.3%), purine metabolism (4.0%), oxidative phosphorylation, (3.5%), starch and sucrose metabolism (3.1%), aminobenoate degradation (2.8%), polycyclic aromatic hydrocarbon degradation (2.3%), bisphenol degradation (2.0%), and others.

Furthermore, the Nt, Nr, Swiss-Prot annotations and KEGG, KOG and GO classifications of 4,857 grain aphid specific unigenes which had no homologous sequences with pea aphid were performed. As indicated in Table 3, less than 15% of these unigenes were predicted with a defined function, whereas the rest of them could not be annotated or classified in the current available public databases, indicating that the functions of these sequences remain unknown and need to be investigated or annotated in the future. In addition, The KEGG classification result showed that among 4,857 grain aphid specific unigenes, 607 unigenes was annotated with KEGG terms which most of them were belonged to the groups of metabolic pathways (33.9%), biosynthesis of secondary metabolites (13.3%), protein processing in endoplasmic reticulum (4.3%), purine metabolism (4.0%), oxidative phosphorylation (3.5%), starch and sucrose metabolism (3.1%), aminobenzoate degradation (2.8%), naphthalene degradation (2.5%), ploycyclic aromatic hydrocarbon degradation (2.3%), bisphenol degradation (2.0%), chlorocyclohexane and chlorobenzene degradation (2.0%) and so forth (Figure 3, See Additional file 3). Given the differences of the host plants and the habitats of these two aphid species, we proposed that the above mentioned pathways might play an important role in biological variations and divergences between pea aphid and grain aphid.

**Table 3:** **Annotations of the 4,857 grain aphid specific unigenes which had no orthologs in pea aphid**

Public databases for annotation	Annotated unigenes	Percentage (%)
Nt	205	4.2
Nr	442	9.1
Swiss-Prot	722	14.9
GO	161	3.3
KOG	530	10.9
KEGG	607	12.5

### The sequence divergences of orthologs between grain and pea aphids

Except for the identified distinct grain aphid and pea aphid specific sequences, the sequence divergences inside the orthologous gene pairs between grain aphid and pea aphids were also estimated. Among 26,667 unigenes homologous to pea aphid mRNA sequences, in total 17,614 sequences pairs were assigned as orthologs. For the 17,614 orthologous gene pairs identified between grain aphid and pea aphid, the CDS of each unigene was obtained by removing 5′ untranslated regions (UTRs) and 3′ UTRs sequence and aligned separately to its counterpart mRNA sequence of pea aphid using a MegaBlast algorithm to generate matched orthologous pairs. The errors caused in the orthologous pair alignment were checked and removed manually. Then, the orthologous pairs below 150 base pairs were also removed in order to generate reasonable results. Finally, 4,191 pairs with CDS larger than 150 base pairs obtained and subjected to KaKs Calculator analysis [15], of these, only 3,368 orthologous pairs could be calculated with non-synonymous (Ka), synonymous (Ks) and their substitution ratio (Ka/Ks).

The sequence divergences within 3,368 orthologous pairs between the two aphid species, of which ratios Ka/Ks substitution could be calculated, had been assessed. For the 5′UTR, the GC content was 38.29% and 4.93% of the compared nucleotides were in a CpG contexts (Table 4). Differences between 5′UTRs of the analyzed orthologous genes of grain aphid and pea aphid occurred at 21.29% of the positions. Interestingly, CpG sites in the 5′UTR differed at 10.94% of positions, whereas non-CpG sites differed at 21.31%. Thus, within 5′UTRs, differences occurred approximately 2 times more often at non-CpG sites than at CpG sites. For the 3′UTR, the GC content was 26.90% and 1.04% of the nucleotides was in a CpG context. The overall difference of 3′UTR between these two aphid species was 18.96%. CpG and non-CpG sites differed at 32.43% and 18.93%, respectively. Hence, in the 3′UTR, CpG sites contained more differences than non-CpG sites. To understand the mechanism of evolution, we compared the ratio of transition (ts) and transversion (tv) [16, 17]. Overall, the transitional differences were more frequent than transversional differences in 5′UTRs and 3′ UTRs (Table 4). Interestingly, the ts/tv ratio was higher in the CpG positions (~1.40) than the non-CpG positions (~1.10) in both the 5′UTRs and 3′ UTRs. When comparing divergences of 3′UTR and 5′UTR, the CpG sites divergence of 3′UTR (1.46) was higher than that of 5′UTR (1.35); however, the overall and non-CpG sites divergence of 5′UTR (1.14, 1.14) was higher than that of 3′UTR (1.12, 1.12) (Table 4).

**Table 4:** **Sequence divergences within orthologous gene pairs between the grain aphid and pea aphid**

	% CpG	% GC	Differences (%)		Compared (kb)	ts/tv ^*****^
			Mean	SE		
5′UTR^*^	4.93	38.29				
ALL			21.29	15.44	638.45	1.14
Non CpG			21.31	15.42	606.96	1.14
CpG			10.94	13.71	31.49	1.35
CDS^**^	15.61	41.47				
ALL			10.53	15.56	4713.66	0.97
Non CpG			11.37	16.61	3977.8	1.00
CpG	.		13.32	19.93	735.86	0.89
nd sites^***^	15.71	45.04				
ALL			8.30	15.30	3009.53	0.72
Non CpG			9.10	16.24	2536.70	0.74
CpG			10.74	19.78	472.83	0.65
4d sites^****^	19.88	37.20				
ALL			16.08	16.72	652.40	1.03
Non CpG			17.03	18.23	522.67	1.03
CpG			20.25	20.67	129.73	1.03
3′UTR	1.04	26.90				
ALL			18.96	14.53	905.70	1.12
non CpG			18.93	14.49	896.27	1.12
CpG			32.43	19.81	9.43	1.46

* UTRs: untranslated regions.

**CDS: coding sequence.

***nd sites: non-degenerative sites.

****4d sites where no changes cause any amino acid replacement.

*****ratio of transitions (ts) over transversions (tv)

Note: The total loci of orthologous gene pairs examined between these two aphid species is 3368.

Among the 3,368 orthologous gene pairs, the overall divergence in coding regions was 10.53%. At non-CpG sites, the divergence was 11.37%, whereas at the CpG sites, the divergence was 13.32%. Apart from CpG context, the nucleotide variations in coding regions could further be classified as non-degenerative (nd) sites (any nucleotide substitutions produced amino acid changes) and four fold degenerate (4d) sites (nucleotide substitutions produced no amino acid changes). From a total of 4,713.66 kb of coding region sequences (CDSs), 3,009.53 kb were nd sites, whereas 652.4 kb were 4d sites (Table 4). At nd sites, the overall divergence was 8.3%, whereas the overall divergence at 4d sites was 16.08%. At nd sites, the GC content was 45.04% and the CpG content was 15.71%. Among the divergence happened at nd sites, the non-CpG sites divergence between two aphid species was 9.10% and the divergence at CpG sites was 10.74%. At the 4d sites, the GC content was 37.20% and the CpG content was 19.88%. Among the divergence happened at 4d sites, the divergence was 20.25% at CpG sites while it was 17.03% at non-CpG sites. These results demonstrated that the higher percentage of divergence at 4d sites compared with nd site was probably proportional to the content of CpG sites (19.88% vs. 15.71%) and the rate of mutation (ts/tv). Comparison the transition and transvertion ratio of nd sites and 4d sites indicated that the transvertional differences were more frequent at nd sites than at 4d sites (Table 4).

Furthermore, the code usages of these 3,368 orthologous pairs between two aphid species were also analyzed by using cusp software (Alan Bleasby, ableasby@hgmp.mrc.ac.uk). The GC content of the orthologous CDSs in grain aphid was 41.66% and that in pea aphid was 39.74%. The GC contents in the first, the second and the third base of triplet-codes in grain aphid were 47.78%, 39.38% and 37.81%, respectively. However, for its counterpart, pea aphid, these values were 46.81%, 38.20% and 34.20%. It was interesting to note that the first base of triplet-codes in both aphid species had highest frequency of G/C usage (or GC content) followed by second base and then the third base.

### Estimation of the substitution rates based on synonymous and non-synonymous analysis between grain aphid and pea aphid

**Figure 4:**
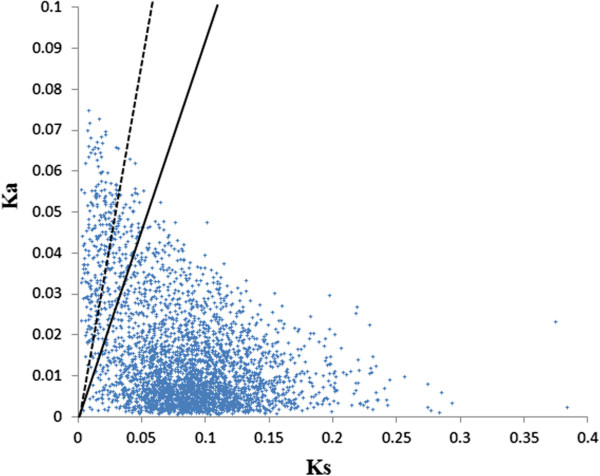
**Distribution of Ka and Ks between orthologs in grain aphid and pea aphid.** Sequences with Ka/Ks > 2 fall above the dash line; while sequences with Ka/Ks of 1–2 fall between the solid and dash lines; Sequences with Ka/Ks <1 below the solid line. Analysis was performed using the method of Yang & Nielsen [21].

Ka/Ks have been widely used to measure the intensity and mode of selection. Ka/Ks > 1 is interpreted as a sign of positive selection whereas Ka/Ks < 1 is a sign of purifying selection [15]. To identify genes undergoing positive and purifying selection, Ka and Ks substitution rates of orthologous pairs between grain and pea aphids were estimated. These 3,368 orthologous gene pairs had mean values of Ka, Ks, and Ka/Ks of 0.016, 0.085 and 0.45, respectively. Of these 3,368 sequence pairs, 179 orthologous gene pairs had Ka/Ks values larger than 2,161 had Ka/Ks values between 1 to 2, and 229 had Ka/Ks values between 0.5 and 1, and the rest 2,799 had Ka/Ks values below 0.5 (See Additional file 4). The distribution of Ka and Ks was as shown in Figure 4 and the top 20 orthologs which had values of Ka/Ks > 1 was as listed in Table 5.

**Table 5:** **The top 20 orthologous unigenes that had Ka/Ks value larger than 1**

Orthology	Ka/Ks	KEGG Annotation	Nr Annotation
**aphid_c7476**	16.77	-	PREDICTED: similar to hormone-sensitive lipase
**aphid_c1598**	14.32	-	PREDICTED: similar to E1a binding protein P400
**aphid_c6800**	13.51	-	PREDICTED: similar to dusky CG9355-PA
**aphid_c28378**	12.64	-	PREDICTED: similar to sugar transporter, partial
**contig01882**	12.11	-	PREDICTED: similar to ctl transporter
**aphid_c4692**	11.83	Biosynthesis of secondary metabolites	PREDICTED: similar to CG9674 CG9674-PA
**aphid_c20070**	11.58	Cell adhesion molecules (CAMs)	Tyrosine-protein phosphatase Lar [Harpegnathos saltator]
**aphid_c9523**	10.65	-	PREDICTED: hypothetical protein LOC100163122, transcript variant 2
**aphid_c381**	10.45	-	PREDICTED: similar to AGAP012271-PA
**aphid_c11292**	9.95	-	PREDICTED: similar to AGAP011393-PA
**contig00933**	9.42	-	PREDICTED: similar to Zinc/iron regulated transporter-related protein 3 CG6898-PA
**aphid_c9186**	8.90	-	conserved hypothetical protein [Culex quinquefasciatus]
**aphid_c11972**	8.78	Glycine, serine and threonine metabolism	PREDICTED: similar to l-allo-threonine aldolase, partial
**aphid_c26862**	8.71	-	PREDICTED: similar to Sug CG7334-PA
**aphid_c26311**	8.56	-	PREDICTED: similar to ADAM metallopeptidase with thrombospondin type 1 motif, 9 preproprotein, partial
**aphid_c390**	8.07	-	PREDICTED: similar to Srp54 CG4602-PA
**contig03210**	8.04	-	PREDICTED: similar to electron-transfer-flavoprotein beta polypeptide
**aphid_c2978**	7.83	Purine metabolism	PREDICTED: similar to GA18461-PA
**aphid_c2071**	7.66	Notch signaling pathway	PREDICTED: similar to nuclear receptor co-repressor 1
**aphid_c9176**	7.36	Arachidonic acid metabolism	PREDICTED: similar to AGAP010241-PA [Acyrthosiphon pisum]

**Figure 5:**
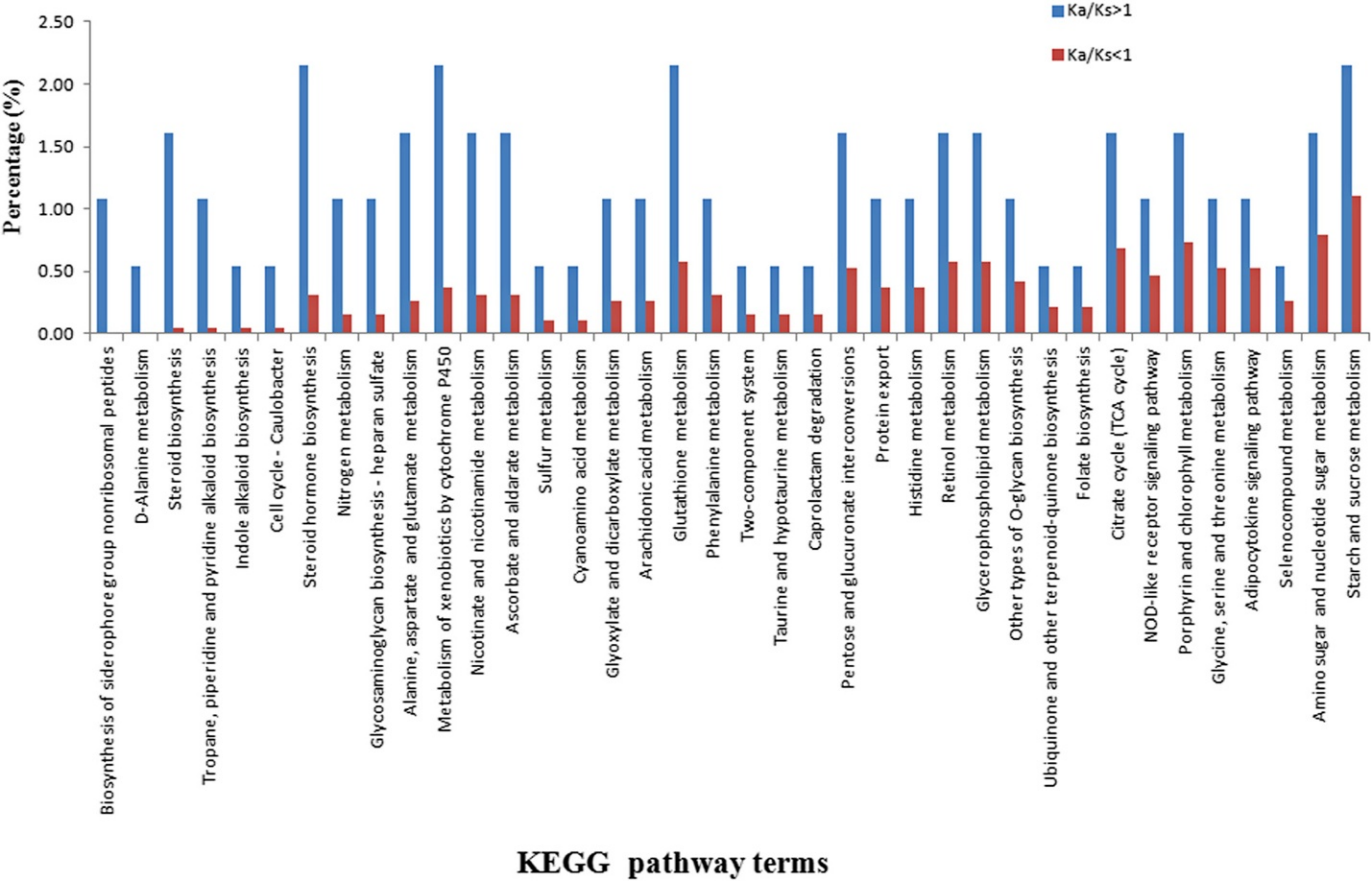
**Comparison of the KEGG pathways of orthologs with ratios of Ka/Ks > 1 and Ka/Ks < 1.** Unigenes involved in pathways such as secondary metabolisms (amino acid, polysaccharide, nucleotide, hormone, sulfur and so forth), detoxification (metabolism of xenobiotics by cytochrome P450), signaling (NOD-like receptor signaling pathway) and so on had higher Ka/Ks values due to positive selection.

**Figure 6:**
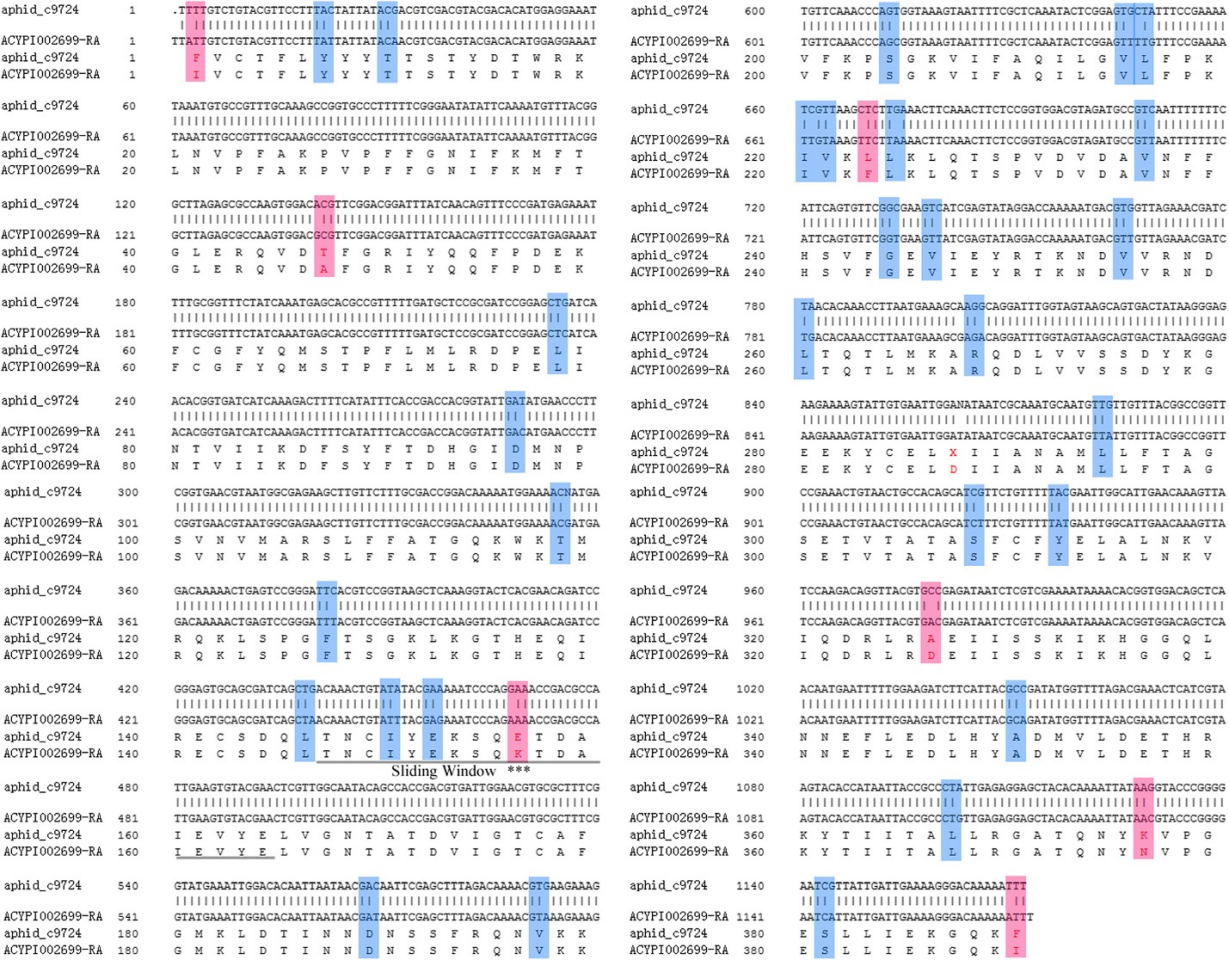
**The DNA and the amino acid sequences alignments of a partial sequence of a cytochrome P450 CYP6AX1 ortholog between grain aphid and pea aphid.** The DNA and amino acid sequences alignments of a cytochorome P450 CYP6AX1 orthologs in grain aphid (aphid_c9724) and pea aphid (ACYPI002699-RA). The Ka/Ks value of this ortholog between the two species is 3.41(p-value of 0.049). The loci shadowed with blue color are synonymous substitutions without amino acid changes, whereas the loci shadowed with pink color are nonsynonymous substitutions with amino acids changes. The amino acid changes without shadowed color indicated ‘not determined’ due to a presence of an ‘N’ in sequencing. The substitution marked with *** is predicted as a locus under positive selection based on KaKs calculation with the sliding window length (underlined) and step length of 57 bp and 6 bp, respectively. (KaKs_Calculator 2.0, Ka/Ks: 1.266).

In addition, of the 3,368 orthologous pairs, 2,088 could be annotated with KEGG terms. According to Ka/Ks values, these 2,088 orthologs could be classified into two groups, which consisted of 186 in one group that had Ka/Ks > 1 and 1,902 in another group had Ka/Ks < 1. The KEGG classification of two groups which had Ka/Ks values <1 and >1 had similar distribution patterns (See Additional file 5). To identify the KEGG pathways which had more unigenes with Ka/Ks value larger than 1, we compared the KEGG classifications of the two groups. The pathways such as secondary metabolisms (amino acid, polysaccharide, nucleotide, hormone, sulfur and so forth), detoxification (metabolism of xenobiotics by cytochrome P450), nucleotide-binding oligomerization domain (NOD)-like receptor signaling pathway and so on had high percentage of unigenes with value of Ka/Ks >1 (Figure 5). Given the fact that similar pathways involved were identified with the grain aphid specific unigenes (Figure 3), we proposed that these pathways might be subjected to positive selection pressure during the process of evolution and play an important role in biological variations and divergences of these two aphid species. For example, a cytochrome P450 6a2-like protein (aphid_c9724, ACYPI002699-RA), which is predicted to take part in enhancing resistance to insecticide [18], had a Ka/Ks value larger than 1 (Ka/Ks = 3.41, p-value of 0.049). The differences in partial DNA and amino acid sequences of a cytochrome P450 6a2-like protein ortholog between grain aphid (aphid_c9724) and pea aphid (ACYPI002699-RA) were as indicated in Figure 6. Sequence alignments indicated that among the 36 nucleoid acid mutations detected, 7 were nonsynonymous substitutions leading to amino acid changes. Further Ka/Ks analysis with sliding window length of 57 bp and a step length of 6 bp indicated that a locus where a nucleoid acid change of GAA to AAA which led to an amino acid change of E to K might be under positive selection during evolution (Ka/Ks = 1.266). This evidence further indicated that this cytochrome P450 6a2-like protein gene might be under strong positive selection during the evolution of the grain aphid with the observed nucleotide acids or amino acid residues subjected to these variations underlying biological divergence or host plants specialization.

## Discussion

Natural selection under different ecological and agricultural environments might be the main cause of evolution and divergence among aphid species, and resulted in their biological and host plants differences. Following publication of the first pea aphid genome in February 2010, aphid biology is entering a new era focused on deciphering many of the specialized biological adaptations underlying the role of aphids as plant pests [8]. So far, only a few genes, such as mitochondrial cytochrome oxidase subunit I (mtDNA-COI), were used to study the differentiation and divergences among different populations of one or different aphid species [19]. However, the investigation of individual gene may not provide an accurate insight into the divergence underlying the biological variations and host plant adaptation of the diverse aphid species at a genome-wide scale. Genome information for additional aphid species needs to be obtained to perform the high-resolution comparative genomic and evolutionary analyses [8]. Comparison between complete genome sequence of pea aphid (as a reference gene set) and expressed sequence tag (EST) data from three other species, green peach-potato aphid (*Myzus persicae*), cotton aphid (*Aphis gossypii*) and *Toxoptera citricida* suggested that a number of the genes evolved fast (high ratio of Ka/Ks), including many genes shared by aphids but with no hit in universal proteins (Uniprot) [13]. Furthermore, comparisons of the genome sequence of pea aphid with ESTs of 8 other aphid species were also performed to verify whether the accumulation of deleterious mutations is the reason for loss of sexual reproduction [14]. Yet, no similar works on grain aphid or the transcriptome data of grain aphid were documented so far.

A more robust picture of genomic divergence among these major agricultural important aphid species may be obtained by examining the transcriptome that have been selected in an unbiased way without prior interest in their biological functions or evolutionary histories [20]. Although the transcriptome data depend on the environment of aphids sampled as well as how the genotype has evolved, the transcriptome represents a sample of the spatiotemporally-expressed genome and can be used as an entry into the genome divergence analysis [20]. In this study, we analyzed the divergence of transcriptome sequence between the two aphid species, and similar genes/pathways were identified to be involved in the divergence of two aphid species through both comparative transcriptomic analysis and Ka/Ks analysis. Comparing with mRNA sequences of pea aphid, 4,857 unigenes were regarded to be specifically expressed in grain aphid under the described rearing conditions (Methods), among which could be annotated by KEGG terms, most of them were involved in secondary metabolism pathways, amino acid metabolism, purine and pyrimidine metabolism and detoxification or insecticide resistance (Figure 3). In addition, to estimate the extent to which selection affects protein-coding sequences, the ratios of Ka/Ks were estimated. Ka/Ks ratio is a good indicator of selective pressure and has been used to identify protein coding sequences under positive and purifying selection [21]. Ka/Ks analysis between grain aphid and pea aphid showed that 340 orthologs were identified to have Ka/Ks > 1, which was interpreted as a sign of positive selection. Many of these genes were involved in KEGG pathways such as amino acid biosynthesis and other biomolecular synthesis and metabolism, polysaccharide metabolism, insecticide resistance and detoxification, and NOD-like receptor signaling pathway (Figure 5). Given that similar involved pathways were identified with the grain aphid specific unigenes, such as secondary metabolism, purine and pyrimidine metabolism and amino metabolism, detoxification and insecticide resistance, these lines of evidences suggested that the above mentioned biological processes and/or pathways were under strongly positive selection and might play important roles in divergence of biological variation and host plants adaptation during the aphid evolution. Similar processes were also identified to be involved in the divergence between two invasive whitefly cryptic species [22].

In addition, a number of divergent sequences within the orthologous gene pairs might also contribute to the biological differences between the two aphid species. The gene divergence at the 5′UTR (21.29%) was more obvious than that of CDS (10.53%) and 3′UTR regions (18.96%). The ratio of ts/tv can indicate the mechanism of evolution [16]. The frequencies of ts were higher than that of tv both in 5′UTR and 3′ UTR region. However, in the coding region, this frequency was slightly lower (Table 4). This result further indicated that the sequence divergences of these two aphid species in un-coding regions of orthologous gene pairs were mainly caused by transitional difference whereas that of the coding regions was mainly due to the transversional variations, especially at the nd sites in which the change of every base of codon would cause the variation of amino acid sequence.

Although the transcriptome of grain aphid generated in our lab and used in this study might have biases due to the fact that the presence of the transcriptome profile depends on the conditions under which aphids were reared and the different developmental stages of aphids, it did not indeed affect the grain aphid specific sequences and orthologous pairs identified by ETE software using a species overlap score (SOS) of 0.0 because of the following reasons. Firstly, we used aphids of mixed life stages to make the library in order to maximize the likelihood of having a whole set of transcripts under the described rearing condition in this study. Secondly, analysis of the transcriptomic divergence under the same rearing conditions such as in an artificial diet assay may also not appropriate because some genes involved in host plants localization and aphid-plant interaction may not expressed at all. At last, the generated transcriptome was assembled by using the genome sequences of pea aphid as a reference set. Based on this, grain aphid specific sequences and sequence divergences between the orthologous pairs of these aphid species was analyzed and divergences in the orthologous pairs in the coding, 5′UTR and 3′ UTR regions were explored with ETE with SOS of 0.0. ETE algorithm uses the level of species overlap between the two branches of a given node to define a duplication (SOS > 0.0) or a speciation (SOS = 0.0) and this has been successfully applied in comparison of gene repertoires and patterns of evolutionary rates in eight aphid species that differ by reproductive mode [14, 23]. And then 186 orthologs which were predicted to be involved in secondary metabolism, xenobiotic metabolisms and etc. were identified to have evolved in response to positive selection based on Ka and Ks analyses. However, we can’t expect to solve all the problems with one single silver bullet, the revealed genetic divergence between grain aphid and pea aphid remains to be appropriately interpreted in light of their biological studies in the near future.

Nevertheless, despite the high sequence homology between grain aphid and pea aphid and its closer relationship, both the transcriptome divergences and the Ka/Ks analyses demonstrated that grain aphid and pea aphid have diverged substantially. And pathways such as the amino acids and other biomolecular synthesis and metabolism, polysaccharide and saccharide metabolism, insecticide resistance and detoxification, might have been subjected to strongly positive selection and play important roles in biological divergences of these two agriculturally important aphid species.

## Conclusions

Comparative analysis between this set of transcriptome of grain aphid generated in our lab by Roche 454 GS-FLX pyrosequencing and mRNA sequences of pea aphid available in the public databases revealed both grain aphid specific sequences and divergent orthologous sequences. And a couple of orthologous genes and/or pathways were identified to have evolved under positive selection pressure which might play important roles in the divergences of grain aphid and pea aphid. To our knowledge, this is the first attempt to study the transcriptome divergence of these two agriculturally important pest species. Our results will provide valuable resources for post-genomic studies of fundamental biological questions in both grain and pea aphids, and investigation of the molecular mechanisms underlying the evolution and divergence of these two destructive aphid species.

## Methods

### Materials

Apterous adult grain aphids derived from a single clonal lineage reared on wheat (*Triticum aestivum* L cv Kenong 199) seedlings were placed in cages for 24 h to produce nymphs. Ten neonate nymphs produced in the 24 h period were transferred to fresh wheat plants. 12 days later, the offspring of these aphids, were selected and subjected to transcriptomic sequencing experiment. Then, 300 grain aphids at different development stages, for example, 60 first instars, 60 second instars, 60 third instars, 60 fourth instars and 60 adults were collected from wheat seedlings with a brush and immediately frozen in liquid nitrogen, and stored at -80°C for RNA extraction.

### RNA sequencing

Total RNA was isolated using a Qiagen RNA Extraction kit according to the manufacturer’s instructions (New England BioLabs). It was treated with RNase-free DNase I for 30 min at 37°C to remove residual DNA, and mRNA was isolated from DNA-free total RNA using a PolyA Tract Kit (Promega, USA) according to the manufacturer’s instructions. In total 500 ng of mRNA isolated from the total RNA, was fragmented by ZnCl_2_ solution, and then purified and condensed with RNeasy MinElute RNA Cleaning up Kit (Qiagen, Germany). The fragmented RNA was eluted from the membrane of the spin tube with 10 μl of RNase-free water, and reverse transcribed into first strand cDNA with a random primer and AMV reverse transcriptase. The second strand of cDNA was synthesized by DNA polymerase I, RNase H and ligase enzyme mixtures. After being blunted and appended with an Adenine base overhang at the 3′ end, the double-stranded cDNA were ligated with GS-FLX sequencing adaptors. The fragments shorter than 500 bp were removed by Ampure Beads according to the manufacturer’s instructions. We used a fluorescence photometer to titer the library and a High Sensitivity DNA Analysis Chip kit to verify the length distribution of the cDNA library.

The small volume (SV) and large volume (LV) emPCRs were carried out according to emPCR kit manual. The enriched beads were counted with a Beckman Coulter Z1, and 2,000,000 beads were loaded into each of 1/2 Pico-Plate region. After the beads were loaded into Pico-Plate, the sequencing reaction was carried out with the parameters of two regions, XL70Li and 200 cycles in a GS-FLX sequencing machine. The image processing and base calling were processed on a 454 data process cluster, automatically.

### Data assembly and analysis

All the raw sequences were then processed to remove low quality and adaptor sequences by using programs such as TagDust [24], LUCY [25] and SeqClean [26] with default parameters. The resulting sequences were then screened against the NCBI UniVec database (http://www.ncbi.nlm.nih.gov/VecScreen/UniVec.html, version 20101122) to remove possible vector sequence contamination. Sequences shorter than 50 bp were discarded. The cleaned data were denovo assembled using Mira 3 with the parameter of “denovo”, “accurate” and “est” (mira --project = aphid--job = denovo, est, accurate,454--fasta = aphid.454.fasta 454_SETTINGS-CL:qc = no.) [27]. The resulting contigs and singletons that were more than 100 bp were retained. To overcome the under-assembled problem of Mira 3 assembler and enhance the quality of assembling, the unigenes were reassembled using gsAssembler v2.3 with an identity parameter of 95% (Roche NimbleGen, Inc., Madison, WI, USA). To eliminate trace contamination of *Buchnera aphidicola* transcripts, which may be introduced due to the miss-paired sequences to oligo(T)_20_ probe using PolyA Tract Kit during mRNA separation, the re-assembled data were filtered by searching against genome sequence of *Buchnera aphidicola* (GenBank no: BA000003.2) using BLAST with a cutoff E-value of 1E-10 [28]. BLAST against public available databases was performed to annotate the functions of these unigenes by using E-value cutoffs of 1E-10, 1E-5 and 1E-5 for Nt, Nr and Swiss-Prot, respectively [29, 30]. The databases used for functional annotation included Nr (http://www.ncbi.nlm.nih.gov; version 20101011), Nt (http://www.ncbi.nlm.nih.gov, version 20101011), and Swiss-Prot (http://www.ebi.ac.uk/uniprot, version 20090819). Moreover, functional classifications were also conducted including Gene Ontology (GO, http://geneontology.org/), KOG (http://www.ncbi.nlm.nih.gov/COG/) and the KEGG pathway (http://www.genome.jp/kegg). GO annotations were conducted by searching against the Nr database using Blast2GO (E-value of 1E-6) [31]. EGO and a custom script were used for assignment of each GO ID to the related ontology entries [32]. KOG and KEGG classifications were performed using BLAST (E-value of 1E-6). ORF analyses were performed by ORF finder (http://www.ncbi.nlm.nih.gov/gorf/gorf.html).

### Synonymous and non-synonymous analysis

The CDS of each sequences of grain aphid were predicted by ORF finder, and then extracted according to the predicted results by a personal perl script written for this purpose. Orthologous relationships between grain aphid and pea aphid genes were conducted by using ETE [23]. We performed a BLAST against predicted genes in pea aphid (BlASTn, a cut-off E-value of 1E-10) for each of the unigene of grain aphid. The sequences aligned with a continuous region longer than 50% of the query sequence were retained and aligned using Muscle 3.8 [33]. All columns in the sequences alignments with gaps were removed using trimAL [34] (http://trimal.cgenomics.org/). Then, the orthologous gene pairs between two aphid species were inferred using the species overlap algorithm described in ETE by using a species overlap score (SOS) of 0.0 [23]. At the same time, we checked the alignments manually to ensure these orthologs were true ones rather than the artifacts of EST assembly.

Coding sequences (CDS) of the orthologous genes were determined using BLASTx software and the CDS sequence of each unigene was extracted using a perl script written for this purpose. The perl script for this analysis was as indicated in Additional file 6. Orthologs were matched by MegaBLAST and orthologous pairs longer than 150 bp were retained. Then, substitution rates of these orthologous genes between grain and pea aphid were estimated separately for synonymous (Ks) and non-synonymous sites (Ka) using an approximate method implemented in the software KaKs Calculator Version 1.2 [15]. Pair-wise approximate analysis were performed using the YN method [21]. As the sequencing errors were distributed among synonymous and non-synonymous sites at equal frequencies, they were not expected to influence the results of analyses [35].

### The GC contents and CpG analysis

GC contents of unigenes, 5′UTRs, 3′ UTRs and CDSs were generated by a perl script written for this purpose (See Additional file 6). The sequence divergence was calculated by dividing the number of substitutions by the number of base pairs compared. The divergence was determined for the contexts of non-degenerate (nd), fourfold degenerate (4d), CpG and non-CpG [36]. The code usages of orthologous gene pairs were analyzed using cusp software (Alan Bleasby, ableasby@hgmp.mrc.ac.uk).

## Electronic supplementary material

Additional file 1: **The flow chart of the assembly of the grain aphid transcriptome data.** (TIFF 2 MB)

Additional file 2: **Annotations and the classifications of the assembled unigenes of grain aphid.** (XLSX 4 MB)

Additional file 3: **The KEGG pathway classifications grain aphid specific unigenes.** (XLSX 2 MB)

Additional file 4: **The Ka, Ks and Ka/Ks values of the orthologous gene pairs between grain aphid and pea aphid.** (XLSX 864 KB)

Additional file 5: **The KEGG pathway classifications of orthologs with the value of Ka/Ks >1 and Ka/Ks <1 between grain aphid and pea aphid.** (XLSX 29 KB)

Additional file 6: **The perl scripts used in this study.** (DOCX 40 KB)

## Abbreviations

BLAST: Basic Local Alignment Search Tool
CDS: Coding Sequence
ESTs: Expressed Sequence Tags
GO: Gene Ontology
KEGG: Kyoto Encyclopedia of Genes and Genomes
KOG: Eukaryotic Orthologous Groups of Proteins
Nr: NCBI Non-redundant Protein Database
Nt: NCBI Nucleotide Acid Database
qPCR: Quantitative Polymerase Chain Reaction
RPKM: Read Per Kb Per Million Bases
RT-PCR: Reverse-Transcript Polymerase Chain Reaction
Uniprot: Universal Protein.
