# Supplementary figures and images for: Comparative transcriptomic analyses revealed divergences of two agriculturally important aphid species
Source: BMC Genomics. 2014 Nov 25;15(1):1023. doi: 10.1186/1471-2164-15-1023 (PMC4301665; doi:10.1186/1471-2164-15-1023)

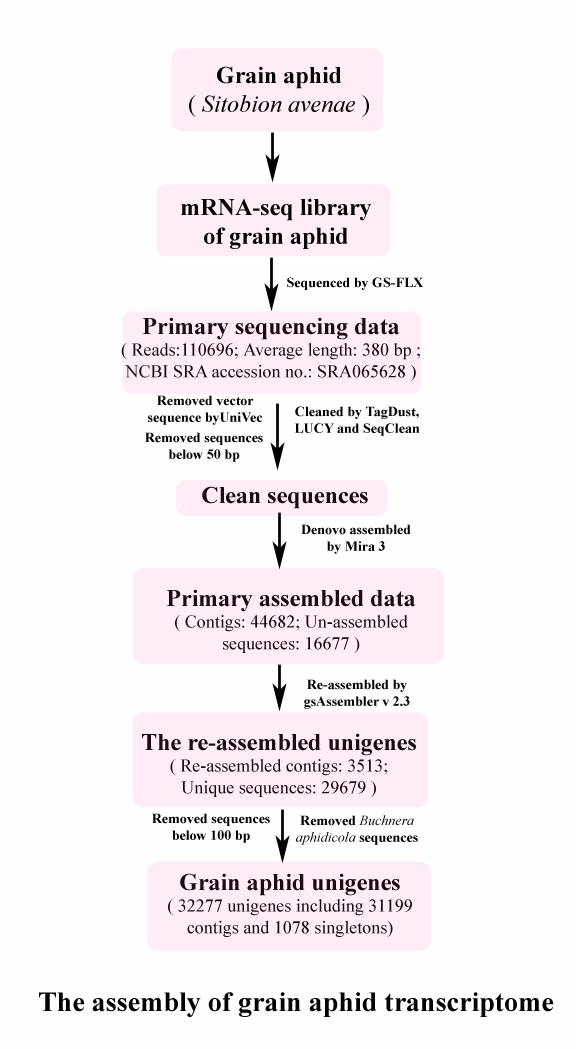

Supplement: Supplementary file 1 — Additional file 1: The flow chart of the assembly of the grain aphid transcriptome data. (TIFF 2 MB) [file 12864_2013_6840_MOESM1_ESM.tiff]
