# Supplementary material for: Comparative transcriptomic analyses revealed divergences of two agriculturally important aphid species
Source: BMC Genomics. 2014 Nov 25;15(1):1023. doi: 10.1186/1471-2164-15-1023 (PMC4301665; doi:10.1186/1471-2164-15-1023)
Supplement: Supplementary file 6 — Additional file 6: The perl scripts used in this study. (DOCX 40 KB) [file 12864_2013_6840_MOESM6_ESM.docx]

1. **#!/usr/bin/perl**

**#==============================================================================**

**# FILE: getOrf.pl**

**#**

**#DESCRIPTION: Extract the coding sequences from orthologous pairs.**

#

#==============================================================================

use strict;

use warnings;

my $seqFile = $ARGV[0] || die "perl $0 <seq_file>\n";

system("getorf -sequence $seqFile -outseq orffinder.result");

my %seq;

open SEQ, "orffinder.result" || die "$!";

$/=">";<SEQ>;$/="\n";

while (<SEQ>)

{ chomp;

my $seqName = $_;

my $unigene_name;

my $starSite;

my $endSite;

if ($seqName =~ /([-\w\.\+]+)_\d+\s+\[(\d+)\s+-\s+(\d+)\]/)

{ $unigene_name = $1;

$starSite = $2;

$endSite = $3;

}

$/ = ">";

my $sequence = <SEQ>;

chomp($sequence);

$sequence =~ s/\s+//g;

$sequence =~ s/\n//g;

if (not exists $seq{$unigene_name})

{ $seq{$unigene_name} = [$starSite,$endSite,$sequence]; }

else {

if (abs($endSite - $starSite) > abs($seq{$unigene_name}[1] - $seq{$unigene_name}[0]))

{ $seq{$unigene_name} = [$starSite,$endSite,$sequence];}

}

$/ = "\n";

}

close SEQ;

open UNIGENE, "$seqFile" || die "$!";

my %unigeneseqs;

$/=">";<UNIGENE>;$/="\n";

while (<UNIGENE>)

{ chomp;

my $seqname = $_;

$/=">";

my $seq = <UNIGENE>;

chomp($seq);

$seq =~ s/\s//g;

$seq =~ s/\n//g;

$unigeneseqs{$seqname} = $seq;

$/="\n";

}

close UNIGENE;

open PRO, ">orf.protein.fa" || die "$!";

open CDS, ">orf.cds.fa" || die "$!";

foreach my $key (keys %seq)

{print $key,"\t",$seq{$key}[0],"\t",$seq{$key}[1],"\t",abs($seq{$key}[1]-$seq{$key}[0])+1,"\t",$seq{$key}[2],"\n";

print PRO ">".$key."\n".$seq{$key}[2],"\n";

my $cdsstart;

my $cdslength;

my $cdsseq;

if ($seq{$key}[0] < $seq{$key}[1])

{ $cdsstart = $seq{$key}[0];

$cdslength = $seq{$key}[1]-$seq{$key}[0]+1;

$cdsseq = substr($unigeneseqs{$key},$cdsstart-1,$cdslength);

}

else

{ $cdsstart = $seq{$key}[1];

$cdslength = $seq{$key}[0]-$seq{$key}[1]+1;

$cdsseq = &revercomple(substr($unigeneseqs{$key},$cdsstart,$cdslength));

}

print CDS ">".$key."\n".$cdsseq,"\n";

}

close PRO;

close CDS;

sub revercomple

{ my $dna = shift;

my $revcom = reverse $dna;

$revcom =~ s/A/T/g;

$revcom =~ s/T/A/g;

$revcom =~ s/G/C/g;

$revcom =~ s/C/G/g;

return $revcom;

}

1. **#!/usr/perl -w**

**#==============================================================================**

**# FILE: mucle_aln_cds_cg_CpG.pl**

**#**

**#DESCRIPTION: Analyze the divergence of orthologous pairs.**

#

#==============================================================================

use strict;

use Bio::AlignIO;

use Bio::Align::AlignI;

use Bio::Align::DNAStatistics;

use Bio::Align::PairwiseStatistics;

use Data::Dumper;

#use Statistics::Basic qw(:all);

use Bio::Tools::Run::Alignment::Clustalw;

use Bio::Align::Utilities qw(aa_to_dna_aln);

use Bio::SeqIO;

use Bio::TreeIO;

use Statistics::Lite qw(:all);

my $aln_fact = Bio::Tools::Run::Alignment::Clustalw->new(-verbose => 0);

my $alignOut = Bio::AlignIO->new(-format=>'clustalw');

my $stats = Bio::Align::DNAStatistics -> new();

my $stats2 = Bio::Align::PairwiseStatistics -> new();

open IN, $ARGV[0] || die "$!";

open IN1, $ARGV[1] || die "$!";

open IN2, $ARGV[2] || die "$!";

my %seqs;

$/=">";<IN>;$/="\n";

while (<IN>)

{ chomp;

my $seqname = $_;

$seqname =~ s/\s+$//;

$/=">";

my $seq = <IN>;

chomp($seq);

$seq =~ s/>//;

$seq =~ s/\n//g;

$seqs{$seqname} = $seq;

$/="\n";

}

close IN;

my %seqs2;

$/=">";<IN1>;$/="\n";

while (<IN1>)

{chomp;

my $seqname = $_;

$seqname =~ s/\s+$//;

$/=">";

my $seq = <IN1>;

chomp($seq);

$seq =~ s/>//;

$seq =~ s/\n//g;

$seqs2{$seqname} = $seq;

$/="\n";

}

close IN1;

my $orthgnecount;

my $cpgnuccount=0;

my @cgratios;

my @sequencebps=(0,0,0);

my @differenceall;

my @differencecpg;

my @differencenocpg;

my @tss=(0,0,0);

my @tvs=(0,0,0);

system("mkdir aligndir") if not -e "aligndir";

while (<IN2>)

{

chomp;

next if (/#/);

next if (/^\s*$/);

$orthgnecount++;

my @tem = split(/\s+/);

my @genes;

if (/ORTHOLOGY RELATIONSHI:/)

{ for(my $i=2; $i<@tem; $i++)

{ push @genes, $tem[$i];}

}

else {for(my $i=0; $i<@tem; $i++) { push @genes, $tem[$i];}

}

next if (@genes > 2);

my %nucs;

my @prots;

open OUT, ">aln.fa" || die "$!";

my $flag = 0;

my $filename="";

foreach my $ele (@genes) {

$filename .= $ele;

if ($ele =~ /ACYP/ and exists $seqs2{$ele})

{#print OUT ">".$ele."\n".$seqs2{$ele}."\n";

my $seqobj = Bio::Seq->new( -display_id => $ele, -seq => $seqs2{$ele});

$nucs{$ele} = $seqobj;

my $protein = $seqobj -> translate();

my $pseq = $protein->seq();

if($pseq =~ /\*/ && $pseq !~ /\*$/)

{

warn("provided a CDS sequence with a stop codon, PAML will choke!");

# exit(0);

}

$pseq =~s/\*//g;

$protein->seq($pseq);

push @prots,$protein;

}

elsif (exists $seqs{$ele})

{

#print OUT ">".$ele."\n".$seqs{$ele}."\n";

my $seqobj = Bio::Seq->new( -display_id => $ele,-seq => $seqs{$ele});

$nucs{$ele} = $seqobj;

my $protein = $seqobj -> translate();

my $pseq = $protein->seq();

if($pseq =~ /\*/ && $pseq !~ /\*$/)

{

warn("provided a CDS sequence with a stop codon, PAML will choke!");

# exit(0);

}

$pseq =~s/\*//g;

$protein->seq($pseq);

push @prots,$protein;

}

else

{

$flag = 1;

last;

}

}

close OUT;

next if ($flag == 1);

if(@prots < 2)

{

warn("Need at least 2 CDS sequences to processed");

exit(0);

}

my $aa_aln = $aln_fact -> align(\@prots);

my $dna_aln = aa_to_dna_aln($aa_aln,\%nucs);

$alignOut -> write_aln($dna_aln);

my $n=0;

my @seq1;

my @seq2;

my $seqname1;

my $seqname2;

my @no_gaps;

foreach my $sequence ($dna_aln->each_seq)

{

$n++;

if ($n == 1) {@seq1 = split(//,$sequence->seq());

$seqname1 = $sequence->display_id();}

if ($n == 2) {@seq2 = split(//,$sequence->seq());

$seqname2 = $sequence->display_id();}

}

for(my $i=0; $i<@seq1; $i++)

{

if ($seq1[$i] ne "-" && $seq2[$i] ne "-")

{ $no_gaps[$i] = 1;}

else { $no_gaps[$i] = 0;}

}

$n = 0;

my $aln_seq1;

my $aln_seq2;

open(OUT,">"."trimed.aln") || die("cannot open output align_output for writing");

for(my $i=0; $i<@no_gaps; $i++)

{

if ($no_gaps[$i] == 1)

{

$n++;

$aln_seq1 .= $seq1[$i];

$aln_seq2 .= $seq2[$i];

}

}

if ($aln_seq1 ne "")

{

print OUT ">".$seqname1."\n".$aln_seq1."\n";

print OUT ">".$seqname2."\n".$aln_seq2."\n";

}

close(OUT);

system("cp aln.fa aligndir/$filename.fa");

system("cp trimed.aln aligndir/$filename.aln");

#system("/home/users/liuqi/tem/wdh/orthlog/trimal-trimAl_1.4/source/trimal -in cds.aln -out trimed.cds.aln -nogaps -fasta");

system("newcpgreport -window 100 -shift 1 -minlen 200 -minoe 0.6 -minpc 50.0 -sequence trimed.aln -outfile trimed.cds.aln.cpg");

system("cp trimed.aln aligndir/$filename.trim.aln");

open CGCP, "trimed.cds.aln.cpg" || die "$!";

my %cpgsites;

my $con = 0;

my $cpgcon = 0;

while (<CGCP>)

{

chomp;

$con++ if (/^ID/);

last if($con == 2);

if(/FT\s+CpG island\s+(\d+)..(\d+)/)

{

$cpgcon = 1;

for (my $i=$1; $i<=$2; $i++)

{

$cpgsites{$i} = 1;

}

}

}

close CGCP;

#if ($cpgcon == 1) {

# $cpggenecount++;

#}

open CPGPART, ">cpgpart.aln" || die "$!";

open NOCPGPART, ">nocpgpart.aln" || die "$!";

open TRIMALN, "trimed.aln" || die "$!";

my $cpgflag = 0;

$/=">";<TRIMALN>;$/="\n";

while (<TRIMALN>)

{ chomp;

my $cpgnuc = "";

my $nocpgnuc = "";

my $seqname = $_;

$/=">";

my $seq = <TRIMALN>;

chomp($seq);

$seq =~ s/\s//g;

$seq =~ s/\n//g;

push @cgratios, &calcgc($seq);

$sequencebps[0] += rindex($seq."\$","\$");

my @seqs = split //,$seq;

for(my $i=0; $i<@seqs; $i++)

{ if(exists $cpgsites{$i+1})

{$cpgnuc .= $seqs[$i];

$sequencebps[1]++;

}

else

{

$nocpgnuc .= $seqs[$i];

$sequencebps[2]++;

}

}

if ($cpgnuc ne "")

{

print CPGPART ">".$seqname."\n".$cpgnuc."\n";

$cpgflag = 1;

}

$cpgnuccount += rindex($cpgnuc."\$","\$");

print NOCPGPART ">".$seqname."\n".$nocpgnuc."\n" if $nocpgnuc ne "";

$/="\n";

}

close TRIMALN;

close CPGPART;

close NOCPGPART;

open OUT, ">aligndir/$filename.stat" || die "$!";

open OUT2, ">>statistics.txt" || die "$!";

my $in1 = new Bio::AlignIO(-format => 'fasta', -file => 'trimed.aln');

my $aln1 = $in1->next_aln;

my $transversions1 = $stats->transversions($aln1);

my $transitions1 = $stats->transitions($aln1);

my $nc1 = $stats2->number_of_comparable_bases($aln1);

my $nd1 = $stats2->number_of_differences($aln1);

$tss[0] += $transitions1;

$tvs[0] += $transversions1;

push @differenceall, $nd1*100/$nc1;

print OUT $transversions1,"\t",$transitions1,"\t",$nc1,"\t",$nd1,"\n";

print OUT2 "$filename"."\t".$nd1*100/$nc1,"\n";

if ($cpgflag == 1) {

my $in2 = new Bio::AlignIO(-format => 'fasta', -file => 'cpgpart.aln');

my $aln2 = $in2->next_aln;

my $transversions2 = $stats->transversions($aln2);

my $transitions2 = $stats->transitions($aln2);

my $nc2 = $stats2->number_of_comparable_bases($aln2);

my $nd2 = $stats2->number_of_differences($aln2);

$tss[1] += $transitions2;

$tvs[1] += $transversions2;

push @differencecpg, $nd2*100/$nc2;

print OUT $transversions2,"\t",$transitions2,"\t",$nc2,"\t",$nd2,"\n";

}

my $in3 = new Bio::AlignIO(-format => 'fasta', -file => 'nocpgpart.aln');

my $aln3 = $in3->next_aln;

my $transversions3 = $stats->transversions($aln3);

my $transitions3 = $stats->transitions($aln3);

my $nc3 = $stats2->number_of_comparable_bases($aln3);

my $nd3 = $stats2->number_of_differences($aln3);

$tss[2] += $transitions3;

$tvs[2] += $transversions3;

push @differencenocpg, $nd3*100/$nc3;

print OUT $transversions3,"\t",$transitions3,"\t",$nc3,"\t",$nd3,"\n";

close OUT;

}

close IN2;

my $cprcount=0;

for my $cpr (@cgratios){ $cprcount += $cpr;}

#my $v1 = vector(@differenceall);

#my $v2 = vector(@differencecpg);

#my $v3 = vector(@differencenocpg);

my $m1 = mean(@differenceall);

my $s1 = stddev(@differenceall);

my $m2 = mean(@differencecpg);

my $s2 = stddev(@differencecpg);

my $m3 = mean(@differencenocpg);

my $s3 = stddev(@differencenocpg);

my $tstv1 = $tss[0]/$tvs[0];

my $tstv2 = $tss[1]/$tvs[1];

my $tstv3 = $tss[2]/$tvs[2];

print $cpgnuccount*100/$sequencebps[0],"\t",$cprcount*100/$sequencebps[0],"\t",$orthgnecount,"\n";

print $m1,"\t",$s1,"\t",$sequencebps[0]/1000,"\t",$tstv1,"\n";

print $m2,"\t",$s2,"\t",$sequencebps[1]/1000,"\t",$tstv2,"\n";

print $m3,"\t",$s3,"\t",$sequencebps[2]/1000,"\t",$tstv3,"\n";

sub calcgc {

my $seq = $_[0];

my @seqarray = split('',$seq);

my $count = 0;

foreach my $base (@seqarray) {

$count++ if $base =~ /[G|C]/i;

}

my $len = $#seqarray+1;

return $count;

}

**3. !/usr/bin/perl**

#==============================================================================

**#**

**# FILE: N50_count.pl**

**#**

**#DESCRIPTION: Calculate the N50 of the assembled unigenes**

#

#

#==============================================================================

use strict;

use warnings;

die "Usage perl $0 seqFile percent_N" if (@ARGV < 2);

my $seqFile = shift @ARGV;

my @percent = @ARGV;

open FI, "fasta.length" || die "$!";

open FO, ">$seqFile.Npercent.statistics.xls" || die "$!";

my %length;

my $total_length;

my $number_reads;

my $max_length=0;

while (<FI>)

{

chomp;

my @tem = split(/\t/);

$total_length += $tem[1];

$number_reads += 1;

$length{$tem[0]} = $tem[1];

$max_length=$tem[1] if $tem[1]>$max_length;

}

close FI;

my %record_read;

my $tem_total;

my %nPercent;

foreach my $key (reverse sort {$length{$a} <=> $length{$b}} keys %length)

{

$tem_total += $length{$key};

foreach my $percent (@percent)

{

if ($tem_total/$total_length >= $percent/100 and (not exists $nPercent{$percent}))

{

print $tem_total/$total_length,"\t",$length{$key},"\n";

$nPercent{$percent} = $length{$key};

}

else

{

$record_read{$length{$key}} = 1;

}

}

}

print FO "Parameter\tLength(bp)\n";

foreach my $percent (sort {$a<=>$b} keys %nPercent)

{

print FO "N$percent ","\t",$nPercent{$percent},"\n";

}

print FO "Average length\t",sprintf("%d",$total_length/$number_reads),"\n";

print FO "Maximum length\t",$max_length,"\n";

print FO "Total sequence number\t",$number_reads,"\n";
